# Effect of the national lifestyle guidance intervention for metabolic syndrome among middle-aged people in Japan

**DOI:** 10.7189/jogh.14.04007

**Published:** 2024-02-09

**Authors:** Yunfei Li, Akira Babazono, Aziz Jamal, Ning Liu, Lifan Liang, Reiko Yamao, Rui Zhao, Lan Yao

**Affiliations:** 1 Department of Epidemiology and Prevention, Center for Clinical Sciences, National Center for Global Health and Medicine, Tokyo, Japan; 2 Department of Health Care Administration & Management, Graduate School of Medical Sciences, Kyushu University, Fukuoka, Japan; 3 Health Administration Program, Faculty of Business & Management, Universiti Teknologi MARA, Selangor, Malaysia; 4 Department of Preventive Medicine and Community Health, University of Occupational and Environmental Health, Kitakyushu, Japan; 5 Department of Human Genetics, University of Chicago, Chicago, Illinois, USA; 6 National Center for Medicine and Technology Assessment, China National Health Development Research Center, Beijing, China; 7 School of Medicine & Health Management, Tongji Medical College, Huazhong University of Science & Technology, Wuhan, China

## Abstract

**Background:**

Japan has implemented a national lifestyle guidance intervention programme for potential metabolic syndrome among adults aged 40–74 years; however, there is limited evidence regarding the causal impact of this intervention. The study aims to determine the causal effect of this intervention on health outcomes and health care utilisation.

**Methods:**

We performed a regression discontinuity design study. A total of 46 975 adults with ≥1 cardiovascular risk factor in 2015 were included in the study. A two-stage evaluation process (stage 1: waist circumference ≥85 cm for men or ≥90 cm for women and ≥1 cardiovascular risk factor; stage 2: body mass index (BMI)≥25 kg/m^2^ and ≥2 cardiovascular risk factors) was applied. Changes in obesity, cardiovascular outcomes, and health care utilisation were evaluated in a one-year follow-up in the fiscal year 2016.

**Results:**

Participants who received lifestyle guidance intervention based on the waist circumference had a statistically significant reduction in obesity outcomes (Δ weight: −0.30 kg, 95% CI = −0.46 to −0.11; Δ waist circumference: −0.26 cm, 95% CI = −0.53 to −0.02; Δ BMI = −0.09 kg/m^2^, 95% CI = −0.17 to −0.04) but not in other cardiovascular risk factors and health care utilisation. Analyses based on BMI and results according to demographic subgroups did not reveal significant findings.

**Conclusions:**

The provision of this intervention had a limited effect on health improvement and a decrease in health care costs, health care visits, and length of stay. A more intensive intervention delivery could potentially improve the efficacy of this intervention programme.

Metabolic syndrome is a cluster of concurrently occurring risk factors [1–3] including obesity, hypertension, hyperglycaemia, and dyslipidaemia, which could increase the risk of cardiovascular disease [4]. The prevalence of metabolic syndrome in adults is estimated to be 20–25% worldwide [5] with 12–37% in the Asian population [6]. An increased health and economic burden of metabolic syndrome has also been reported with its increasing prevalence at both the individual and national levels [7,8]. Metabolic syndrome can be diagnosed at an early stage through health checkups. Timely management and intervention directed at an early stage could substantially reduce the progression of severe morbidity, allowing financial savings and reducing complex household economic burden [9,10].

Recognising the importance of early detection and intervention for metabolic syndrome, Japan implemented a lifestyle guidance intervention programme for adults with metabolic syndrome in 2008. The objectives of this programme are to increase the awareness of metabolic syndrome among the general public, to identify populations at risk, to guide for managing one’s health through modification of lifestyle and behaviours, and to reduce health care spending and the economic burden. It is estimated that over 27 million people in Japan receive annual health checkups, and approximately one million individuals received lifestyle guidance interventions in 2016, with an allocation of over 16 billion Japanese yen (JPY) (JPY 120 = USD 1) to finance this programme [11].

Some studies have evaluated the effectiveness of this lifestyle guidance intervention programme [12–16], however, some issues remain unresolved. First, the limited selection of participants, such as a study that was conducted on one specific type of occupation, limits the generalisability of the study findings. Second, most studies have focused on evaluating the effect of health outcomes; however, the effect on health care utilisation has not been reported in previous studies. In this study, we used a quasi-experimental approach, a regression discontinuity design, to estimate the causal impact of lifestyle guidance intervention on health outcomes and health utilisation after one year using population-based data, including employees from a wide variety of companies. We believe that our findings can help enhance the efficacy of this national intervention programme.

## METHODS

### Data source and sample

We obtained data from the Japanese Health Insurance Association, Fukuoka Branch, which is the largest employment-based health insurance programme in Japan. The anonymised insurance claims data are acquired through a special arrangement between the association and the university’s research department. Although the data are not publicly available, its use for academic research purposes is normally allowed. Interested parties could contact the insurance association for inquiries. Information on annual health checkups of individuals aged 35–74 years is available from this association. We obtained data from two waves: 1) fiscal year (FY) 2015, from 1 April 2015 to 31 March 2016; and 2) FY 2016, from 1 April 2016 to 31 March 2017. Some participants had ≥2 health checkup records every year, and we extracted the first record from each wave. We collected data by linking health checkups and health care claims databases from the association. The health checkup database included participants’ demographic information (age, sex), socioeconomic status (occupation, monthly income), lifestyle and health behaviours (current smoking, alcohol drinking, exercise habits, and dietary habits), anthropometric measurements (waist circumference, weight, and body mass index (BMI)), cardiovascular risk factors (systolic and diastolic blood pressure, total cholesterol, triglyceride, and low-density lipoprotein (LDL) cholesterol levels), and medication use (antihypertensive, antidiabetic, antihyperglycemic, and antihyperlipidemic drugs). The health care claims database includes participants’ information about diagnoses and medical treatment records, as well as health care utilisation. We linked the data by matching each patient’s identification number, sex, and date of birth in the two databases. In this study, we used variables reported in FY 2015 as baseline characteristics and examined the outcome change in FY 2016. For health care costs, we used a conversion rate of JPY 100 = USD 1.

### Health checkups and lifestyle guidance intervention

The health checkup programme is conducted annually. All employed adults aged 40–74 years are mandated to undergo annual health checkups to identify high-risk populations and provide lifestyle guidance to improve their awareness of developing healthy lifestyle behaviours. Health checkups include basic anthropometric measurements, as well as blood pressure and cholesterol testing. A self-report questionnaire is compulsory during health checkups.

The identification of high-risk populations comprised multiple steps. First, individuals who were currently receiving treatment for any chronic disease were advised to receive treatment and guidance from a physician and were automatically excluded from the intervention programme. Second, individuals with abnormal sex-specific waist circumference measurements (≥85 cm for men and ≥90 cm for women) together with one or more cardiovascular risk factors (hypertensive, hyperglycemic, or hyperlipidemic status) were required to receive the intervention. Finally, individuals with BMI≥25 kg/m^2^ and two or more cardiovascular risk factors were also required to undergo the programme.

The selected individuals are required to participate in a support programme that includes basic education on metabolic syndrome, health counseling, physical activity, and healthy dietary habits via interviews, telephone calls, and e-mail. This intervention programme aims to improve awareness of metabolic syndrome through improvements in lifestyle and behaviour. This intervention is usually conducted in collaboration with physicians, public health nurses, and registered dietitians. The process is usually initiated with a preliminary interview conducted by a well-trained instructor, who is usually a qualified public health nurse or dietitian. Participants were then followed up for three to six months. The duration of follow-up depended on the individual’s baseline anthropometric measurements and cardiovascular risk factors (Box S1 in the **Online Supplementary Document**).

### Study design

#### Participants

**Figure 1:**
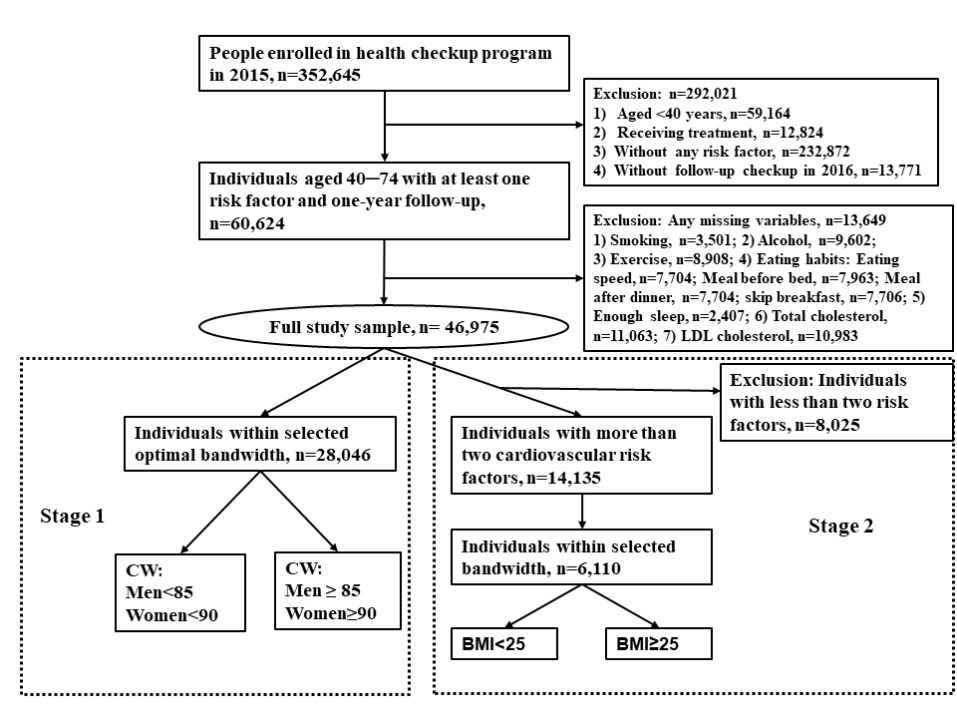
Flowchart of the exclusion and inclusion criteria in the regression discontinuity design. BMI – body mass index, LDL – low-density lipoprotein, WC – waist circumference

Among the 352 645 people who enrolled in the health checkup programme in 2015, people aged ≤40 years, receiving medical treatment, without risk factors, without follow-up information, and those who had missing variables were excluded, leaving 46 975 participants for analysis (**Figure 1**).

A two-stage analysis strategy was used. Stage 1 involved the identification of participants with at least one cardiovascular risk factor in 2015, and stage 2 identified participants with at least two cardiovascular risk factors in 2015. In total, 46 975 individuals with at least one cardiovascular risk factor (stage 1) and 14 135 individuals with at least two cardiovascular risk factors (stage 2) were included in the analysis.

#### Regression discontinuity design

We used a quasi-experimental regression discontinuity design to establish the causal impact of the intervention on health outcomes. An assignment variable was applied by designating a cutoff or threshold above or below an intervention [17]. The assignment variable according to a threshold in regression discontinuity can either be ‘sharp’ or ‘fuzzy’ [17–19]. In a sharp regression discontinuity design, the intervention is deterministically assigned according to the value of the assignment variable. In a fuzzy regression discontinuity design, the intervention is probabilistically determined by the value of the assignment variable, although the probability of being treated is higher on one side of the threshold [18–20]. In this study, we applied a fuzzy regression discontinuity design by the combination of anthropometric measurements (waist circumference and BMI) and cardiovascular risk factors, which was in accordance with the selection criteria of metabolic syndrome in the Japanese guidelines [21]. Because the cutoffs of waist circumference in stage 1 were sex-specific (85 cm for men and 90 cm for women), we introduced a new variable, standardised waist circumference (SWC), which represented the difference between waist circumference and the cutoffs (85 cm for men and 90 cm for women) in 2015. In this study, SWC was used as the assignment variable in stage 1 and BMI was used as the assignment variable in stage 2. The threshold of SWC was set at 0 in stage 1, and the threshold of BMI was set at 25 kg/m^2^ in stage 2.

#### Health outcomes

In this study, the primary outcome was changed in body weight measurements (body weight, waist circumference, and BMI) after one year of lifestyle guidance intervention. Our secondary outcomes were changes in cardiovascular risk factors (systolic and diastolic blood pressure, total cholesterol, triglyceride cholesterol, and LDL cholesterol) and the utilisation of health care services (outpatient visit frequency, outpatient costs, inpatient frequency, inpatient costs, and length of stay) at one year after receiving the national lifestyle guidance intervention.

#### Covariates

Researchers are encouraged to include some covariates whenever possible to improve the precision of the estimates [17,22]. Hence, we included demographic and socioeconomic characteristics and health behaviours as model covariates. Income status, represented by monthly income, was analysed as a continuous variable. Occupation was classified as a binary variable: manual or non-manual labor.

### Analytical approach

We implemented a local linear approach to estimate the impact of lifestyle guidance interventions in 2015 on changes in health outcomes in 2016. Local linear polynomials were chosen over higher order polynomials to prevent overfitting of the data and under- or overestimation [23]. Based on evidence for a potential nonlinear relationship between the assignment variable and outcome, local quadratic regression was added to check the robustness of the model. A triangular kernel function was used to assign more weight to observations near the threshold.

For the main local linear results, we fitted the regression discontinuity models separately for SWC and BMI as shown below.

For SWC in stage 1:

*Y_i_ = α*_0_ *+ α*_1_*Above_i_ + α*_2_ (*SWC_i_ −* 0) *+ α*_3_*Above_i_* (*SWC_i_ −* 0) *+ γX_i_ + ε_i_* ·  ·  ·  ·  ·  ·  ·  · (1)

And for BMI in stage 2:

*Z_j_ = β*_0_ *+ β*_1_*Above_j_ + β*_2_ (*BMI_j_ –* 25) *+ β_3_Above_j_ (BMI_j_ –* 25) *+ δX_j_ + ϕ_j_* ·  ·  ·  ·  ·  ·  ·  · (2)

where is a measure of the change in outcome for individual *i* based on SWC, and *Z_j_* is a measure of the change in outcome for individual *j* based on BMI; *Above_i_* is an indicator variable equal to 1 for people whose SWC is at least 0 (Equation 1), and *Above_j_* is an indicator variable equal to 1 for people whose BMI is at least 25 kg/m^2^ (Equation 2) in 2015; *SWC_i_* is a measure of an individual’s SWC in 2015, and *BMI_j_* is a measure of an individual’s BMI in 2015; *X_i_* and *X_j_* are vectors of covariates in 2015 predicting the outcome. In these models, α_1_ and β_1_ are local average causal impacts of the lifestyle guidance intervention; α_2_ is the marginal change in outcome in 2016 when the SWC in 2015 is lower than 0, and β_2_ is the marginal change in outcome in 2016 when the BMI in 2015 was lower than 25 kg/m^2^; (α_2_ + α_3_) is the marginal change in outcome in 2016 when the SWC in 2015 was at least 0, and β_2_ + β_3_ is the marginal change in outcome in 2016 when the BMI in 2015 was at least 25 kg/m^2^.

Before conducting the regression discontinuity analyses, we examined the applicability of the proposed models. First, we examined the manipulation of the assignment variables (SWC/BMI) based on the McCray test [24] because the regression discontinuity design requires no manipulation of the assignment variables around the threshold [25]. Second, we examined the continuity of individuals’ characteristics around the threshold because the regression discontinuity design requires that no other interventions or changes affecting the outcome be correlated with the threshold. In this study, we examined the mean demographic, socioeconomic, and health behaviour characteristics of people just above and below the thresholds within the selected optimal bandwidths.

The choice of bandwidth is important because estimates with a smaller bandwidth might have a small degree of bias but a high variance, whereas estimates with a wider bandwidth might have a high degree of bias but a low variance [26,27]. We determined the bandwidth around the SWC/BMI thresholds using a data-driven optimal bandwidth method. The optimal bandwidth for waist circumference was 7.8–9.6; therefore, we set a bandwidth of 8.0 cm for SWC. Likewise, we adopted a bandwidth of 2.0, from the BMI threshold, because the optimal bandwidth was 1.9–2.2. We developed several models and conducted regression analyses separately by adding the following: 1) demographic, 2) demographic and socioeconomic, and 3) demographic, socioeconomic, and behavioural covariates. We also evaluated the presence of heterogeneity and its impact on the estimates by performing sub-analyses based on sex and age categories (40–54, 55–64, and 65–74 years).

After estimating the primary and secondary outcomes, we conducted additional regression discontinuity analyses to elucidate potential downstream changes in health behaviours resulting from the lifestyle guidance intervention. Effects on smoking, alcohol consumption, regular exercise, and dietary behaviours were determined. For these binary outcomes, we used a modified Poisson regression approach to estimate the risk ratios [28]. Additionally, we evaluated the sensitivity of our results to the choice of bandwidth by setting a range of potential bandwidths. All statistical analyses in this study were performed using Stata Release 15 (Stata Corp LLP, College Station, TX, USA).

## RESULTS

### Sample characteristics

Of the 352 645 individuals who participated in the national medical checkup programme in 2015, 46 975 were eligible for inclusion in the sample for analysis. **Table 1** shows the characteristics of the total sample and the samples within the selected optimal bandwidths for SWC and BMI. The proportions of demographic, socioeconomic, and baseline health behaviour characteristics were similar for all three samples. The mean age was approximately 58 years, the mean monthly income was approximately USD 3200, around 37% worked in occupations involving manual labor, and approximately 30% of participants were smokers in 2015.

**Table 1:** Demographic, socioeconomic, and health behaviour characteristics for the total sample and samples within the optimal selected bandwidth in 2015

Characteristics	Baseline SWC*		Difference in means (n = 28 046)	Baseline BMI		Difference in means (n = 6110)
	**<0 cm (n = 13 389)**	**≥0 cm (n = 14 657)**		**< 25 (n = 3 105)**	**≥ 25 (n = 3005)**	
**Baseline demographic**						
Mean age, year (SD)	57.9 ± 7.5	57.7 ± 7.6	−0.2	59.6 ± 7.0	58.6 ± 7.2	−1.0
Men, %	70.4	83.2	12.8	77.6	78.0	0.4
**Socioeconomic**						
Income, USD/month	3066.8 ± 2004.9	3359.4 ± 2189.8	292.6	3228.9 ± 2204.1	3297.6 ± 2242.6	68.7
Occupation, %	37.0	37.8	0.8	36.8	35.7	−0.9
**Health behaviour, %**						
Smoke	29.0	30.9	1.9	27.9	28.9	1.0^*^
Drink alcohol	53.6	56.7	3.1	51.8	50.9	−1.1
Regular exercise	28.6	26.2	−2.4	26.3	24.2	−1.9
Dietary habits						
*Normal eating speed*	66.9	63.1	−3.8	67.1	61.7	−5.4
*Supper before bed*	33.3	34.3	1.0	34.1	35.5	1.4
*Supper after dinner*	43.3	47.1	3.8	45.5	50.0	4.5
*Skip breakfast*	27.9	28.8	0.9	30.9	32.8	1.9
Enough sleep	50.7	50.5	−0.2	51.9	48.3	−3.6

BMI – body mass index, LDL – low-density lipoprotein, mm Hg – millimeter of mercury, SD – standard deviation, SWC – standardised waist circumference, USD – US dollar

*SWC for men is 85 cm and 90 cm for women.

### Manipulation of assignment variables

**Figure 2:**
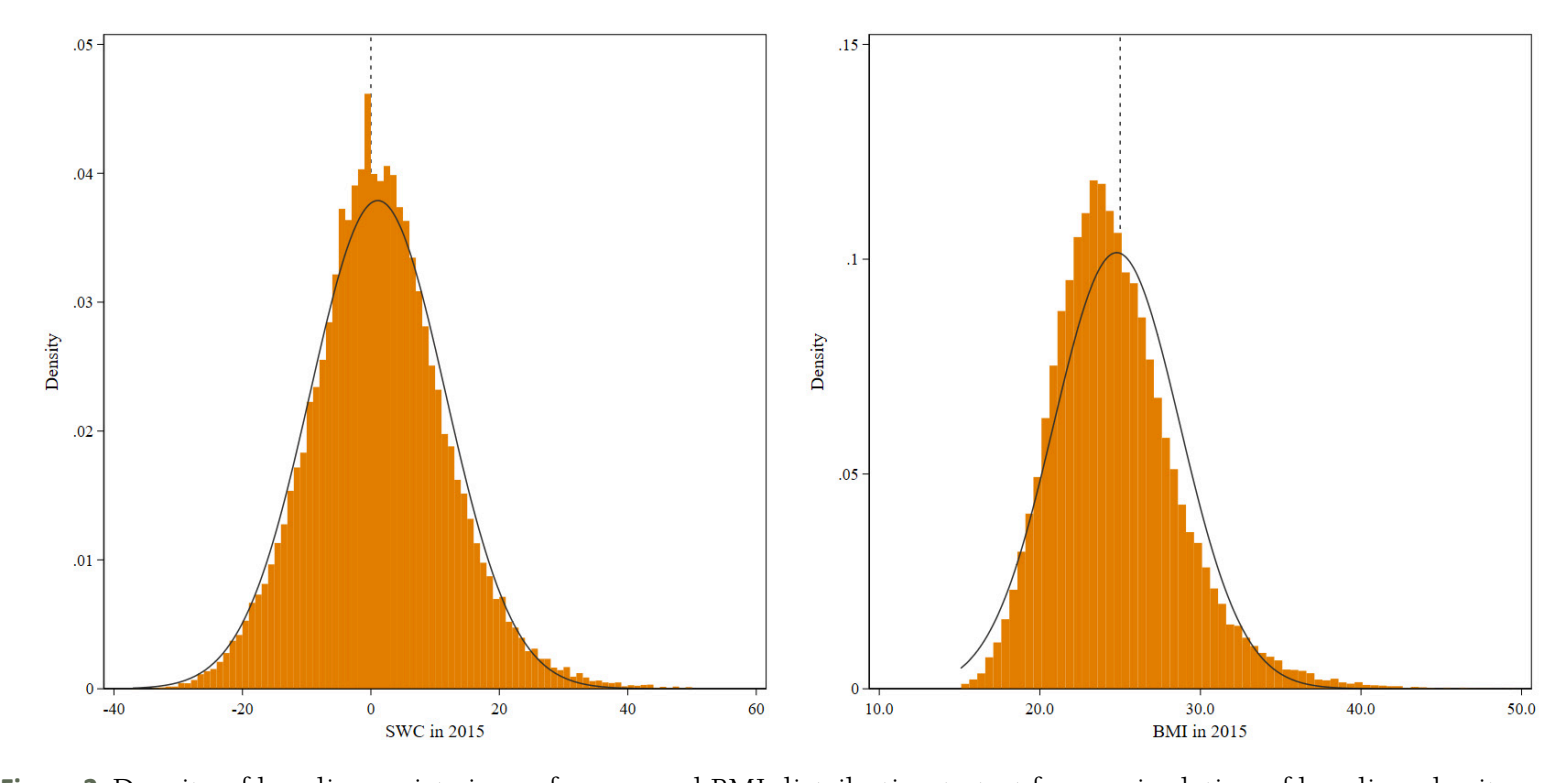
Density of baseline waist circumference and BMI distribution to test for manipulation of baseline obesity status at threshold. The dashed line represents the distance to the cutoffs (men: 85 cm and women: 90 cm) and BMI (25 for both men and women), which was used to identify adults who were strongly encouraged to receive lifestyle guidance intervention to change their lifestyle habits. Sample size = 46 975. BMI – body mass index, SWC – standardised waist circumference

Mean waist circumference was 87.5 cm (SD = 10.1 cm), and mean BMI was 24.8 kg/m^2^ (SD = 4.1 kg/m^2^). We examined the continuity of the assignment variables (SWC and BMI in 2015). The value was 1.16, and the associated *P*-value was 0.055 for SWC. For BMI, the value was 1.18 and the associated *P*-value was 0.545. Thus, under the continuity-based approach, we failed to reject the null hypothesis of no difference in the density of intervention and non-intervention observations at both thresholds (0 for SWC and 25 kg/m^2^ for BMI). **Figure 2** presents a histogram of the assignment variables to visually check for manipulation. There was no evidence of clustering at any of the specified thresholds based on visual inspection.

### Continuity of characteristics around thresholds

The regression discontinuity design requires that no other interventions or changes that could potentially affect the outcome be correlated with the SWC or BMI thresholds. **Table 2** shows the mean demographic, socioeconomic, and health behaviour characteristics for participants just above and below the thresholds within the optimal bandwidths. Most of the characteristics were balanced around both thresholds (SWC and BMI). Smoking was the only slightly unbalanced factor when BMI was used as the assignment variable in stage 2. According to the method of Imbens and Lemieux [29] we observed that this variable was continuous near the threshold after narrowing the bandwidth to 1.0, or widening the bandwidth to 3.0. In our regression analyses, we included results that controlled for these covariates and found that the results were robust.

**Table 2:** Covariate means above and below waist circumference discontinuity thresholds within selected optimal bandwidths in 2015*

Characteristics	Baseline SWC†		Difference in means (n = 28 046)	Baseline BMI†		Difference in means (n = 6110)
	**<0 cm (n = 13 389)**	**≥0 cm (n = 14 657)**		**<25 (n = 3 105)**	**≥25 (n = 3005)**	
**Baseline demographic**						
Mean age, year (SD)	57.9 ± 7.5	57.7 ± 7.6	−0.2	59.6 ± 7.0	58.6 ± 7.2	−1.0
Men, %	70.4	83.2	12.8	77.6	78.0	0.4
**Socioeconomic**						
Income, USD/month	3066.8 ± 2004.9	3359.4 ± 2189.8	292.6	3228.9 ± 2204.1	3297.6 ± 2242.6	68.7
Occupation, %	37.0	37.8	0.8	36.8	35.7	−0.9
**Health behaviour, %**						
Smoke	29.0	30.9	1.9	27.9	28.9	1.0
Drink alcohol	53.6	56.7	3.1	51.8	50.9	−1.1
Regular exercise	28.6	26.2	−2.4	26.3	24.2	−1.9
Dietary habits						
*Normal eating speed*	66.9	63.1	−3.8	67.1	61.7	−5.4
*Supper before bed*	33.3	34.3	1.0	34.1	35.5	1.4
*Supper after dinner*	43.3	47.1	3.8	45.5	50.0	4.5
*Skip breakfast*	27.9	28.8	0.9	30.9	32.8	1.9
Enough sleep	50.7	50.5	−0.2	51.9	48.3	−3.6

BMI – body mass index, SD – standard deviation, SWC – standardised waist circumference, USD – US dollar

*Bandwidth is −8 to 8 cm for SWC and 23 to 27 for BMI. SWC is the distance to the cutoffs (men, 85 cm; women, 90 cm).

†To test the smooth continuity of the observed covariates at the threshold of SWC/BMI, we followed the methods of Lee et al. and Imbens and Lemiux. We applied a regression discontinuity design, using covariates as the outcome variable and SWC/BMI as the explanatory variable. Significant discontinuity was observed when smoking was used as the outcome variable and BMI was used as the assignment variable in Stage 2. However, the validity of the design still holds because we found that this variable was continuous near the threshold after narrowing the bandwidth to 1 or widening the bandwidth to 3. We did not observe any discontinuity in the other observed covariates at the threshold of SWC/BMI.

### Impact of lifestyle guidance intervention

**Table 3** presents the results of the regression discontinuity estimates using the local linear approach, demonstrating the impact of the lifestyle guidance intervention. We found that the intervention based on health checkups using waist circumference criteria in 2015 caused a significant reduction in obesity outcomes in 2016. The impact size of this intervention did not differ when we controlled for demographic, socioeconomic, and behavioural covariates. We failed to observe a significant change in cardiovascular or health care utilisation outcomes. Additionally, lifestyle guidance intervention based on health checkups using BMI criteria in 2015 caused a significant reduction in diastolic blood pressure and inpatient costs when adjusted for demographic, socioeconomic, and behavioural covariates.

**Table 3:** Regression discontinuity estimates (95% confidence intervals) of the impact of lifestyle guidance intervention in 2015 on changes in health outcomes and health utilisation within one year, fitted using local linear regression*

	SWC				BMI			
**Impact of lifestyle guidance intervention**	**Without covariates**	**With demographic covariates**	**With demographic and socioeconomic covariates**	**With demographic, socioeconomic, and behavioural covariates**	**Without covariates**	**With demographic covariates**	**With demographic and socioeconomic covariates**	**With demographic, socioeconomic, and behavioural covariates**
**Obesity outcomes**								
Δ† Weight	−0.30 (−0.47 to −0.15, *P* < 0.001)	−0.30 (−0.46 to −0.15, *P* < 0.001)	−0.30 (−0.46 to −0.15, *P* < 0.001)	−0.30 (−0.46 to −0.11, *P* = 0.001)	0.19 (−0.28 to 0.43, *P* = 0.680)	0.9 (−0.28 to 0.43, *P* = 0.669)	0.19 (−0.28 to 0.43, *P* = 0.679)	0.05 (−0.10 to 0.19, *P* = 0.523)
Δ† Waist circumference	−0.26 (−0.52 to −0.06, *P* = 0.009)	−0.26 (−0.52 to −0.06, *P* = 0.013)	−0.26 (−0.52 to −0.06, *P* = 0.013)	−0.26 (−0.53 to −0.02, *P* = 0.031)	0.18 (−0.38 to 0.63, *P* = 0.619)	0.18 (−0.37 to 0.64, *P* = 0.595)	0.18 (−0.37 to 0.64, *P* = 0.599)	0.04 (−0.56 to 0.58, *P* = 0.971)
Δ† BMI	−0.10 (−0.17 to −0.05, *P* < 0.001)	−0.10 (−0.17 to −0.05, *P* < 0.001)	−0.10 (−0.17 to −0.05, *P* < 0.001)	−0.09 (−0.17 to −0.04, *P* = 0.002)	0.06 (−0.10 to 0.16, *P* = 0.626)	0.06 (−0.09 to 0.16, *P* = 0.617)	0.06 (−0.10 to 0.16, *P* = 0.628)	0.05 (−0.10 to 0.19, *P* = 0.523)
**Cardiovascular outcomes**								
Δ† Systolic blood pressure	−0.29 (−1.38 to 0.81, *P* = 0.610)	−0.28 (−1.37 to 0.82, *P* = 0.621)	−0.28 (−1.37 to 0.81, *P* = 0.619)	−0.34 (−1.75 to 0.61, *P* = 0.610)	−0.95 (−3.60 to 1.51, *P* = 0.422)	−0.95 (−3.59 to 1.51, *P* = 0.423)	−0.95 (−3.59 to 1.51, *P* = 0.423)	−1.67 (−5.03 to 0.58, *P* = 0.120)
Δ† Diastolic blood pressure	−0.09 (−0.64 to 0.78, *P* = 0.850)	−0.08 (−0.63 to 0.79, *P* = 0.826)	−0.09 (−0.63 to 0.79, *P* = 0.832)	−0.14 (−0.83 to 0.71. *P* = 0.869)	−0.06 (−2.52 to 0.87, *P* = 0.342)	−0.06 (−2.52 to 0.88, *P* = 0.343)	−0.06 (−2.53 to 0.87, *P* = 0.338)	−0.87 (−3.94 to −0.21, *P* = 0.029)
Δ† Total cholesterol	−0.53 (−2.63 to 1.27, *P* = 0.493)	−0.54 (–2.65 to 1.25, *P* = 0.481)	−0.55 (−2.66 to 1.23, *P* = 0.472)	−1.32 (−3.23 to 0.97, *P* = 0.292)	−0.96 (−7.48 to 1.32, *P* = 0.170)	−0.99 (−7.52 to 1.27, *P* = 0.163)	−0.96 (−7.48 to 1.30, *P* = 0.168)	−0.19 (−7.36 to 2.37, *P* = 0.315)
Δ† Triglyceride	−0.64 (−9.63 to 5.19, *P* = 0.557)	−0.61 (−9.59 to 5.23, *P* = 0.564)	−0.64 (−9.62 to 5.20, *P* = 0.559)	1.32 (−8.16 to 8.18, *P* = 0.998)	2.89 (−16.56 to 21.18, *P* = 0.810)	2.97 (−16.43 to 21.30, *P* = 0.800)	3.00 (−16.39 to 21.35, *P* = 0.797)	1.69 (−20.87 to 23.78, *P* = 0.898)
Δ† LDL cholesterol	−0.09 (−1.68 to 1.79, *P* = 0.951)	−0.11 (−1.70 to 1.77, *P* = 0.967)	−0.11 (−1.71 to 1.76, *P* = 0.977)	−0.60 (−2.53 to 1.24, *P* = 0.505)	−0.62 (−6.49 to 1.36, *P* = 0.201)	−0.65 (−6.53 to 1.31, *P* = 0.193)	−0.63 (−6.50 to 1.34, *P* = 0.197)	−0.16 (−6.34 to 2.36, *P* = 0.370)
**Healthcare utilisation outcomes**								
Δ† Outpatient frequency	0.57 (−1.39 to 1.49, *P* = 0.948)	0.49 (−1.49 to 1.37, *P* = 0.934)	0.53 (−1.45 to 1.41, *P* = 0.980)	0.56 (−1.33 to 1.85, *P* = 0.751)	−1.04 (−4.89 to 0.35, *P* = 0.089)	−1.03 (−4.90 to 0.33, *P* = 0.086)	−1.12 (−4.97to 0.24, *P* = 0.075)	−1.09 (−5.10 to 0.92, *P* = 0.174)
Δ† Outpatient cost	74.8 (−146.5 to 337.7, *P* = 0.439)	72.0 (−150.5 to 333.5, *P* = 0.458)	73.2 (−148.8 to 335.0, *P* = 0.451)	66.1 (−191.5 to 348.9, *P* = 0.568)	−38.5 (−732.4 to 183.1, *P* = 0.240)	−39.2 (−733.7 to 181.5, *P* = 0.237)	−36.4 (−730.5 to 184.4, *P* = 0.242)	−32.1 (−872.8 to 244.5, *P* = 0.270)
Δ† Inpatient frequency	0.02 (−0.07 to 0.10, *P* = 0.748)	0.02 (−0.07 to 0.10, *P* = 0.762)	0.02 (−0.07 to 0.10, *P* = 0.765)	−0.01 (−0.10 to 0.09, *P* = 0.897)	−0.04 (−0.26 to 0.13, *P* = 0.537)	−0.04 (−0.26 to 0.13, *P* = 0.523)	−0.04 (−0.26 to 0.13, *P* = 0.528)	−0.05 (−0.33 to 0.14, *P* = 0.422)
Δ† Inpatient cost	123.2 (−434.8 to 802.6, *P* = 0.560)	121.1 (−437.9 to 800.0, *P* = 0.567)	119.9 (−440.0 to 797.4, *P* = 0.571)	−1.2 (−549.3 to 804.3, *P* = 0.712)	−633.2 (−2832.6 to 4.2, *P* = 0.051)	−642.1 (−2848.3 to −10.2, *P* = 0.048)	−643.8 (−2849.4 to −11.3, *P* = 0.048)	−782.2 (−3499.5 to −83.8, *P* = 0.040)
Δ† Length of stay	0.39 (−0.52 to 1.50, *P* = 0.338)	0.38 (−0.52 to 1.50, *P* = 0.346)	0.38 (−0.52 to 1.49, *P* = 0.346)	0.07 (−0.86 to 1.37, *P* = 0.654)	−1.10 (−4.37 to 2.58, *P* = 0.614)	−1.10 (−4.39 to 2.57, *P* = 0.609)	−1.13 (−4.41 to 2.55, *P* = 0.599)	−1.21 (−5.33 to 3.15, *P* = 0.615)

SWC – standardised waist circumference, BMI – body mass index, LDL – low-density lipoprotein

*The sample comprised people within the optimal bandwidth of the threshold of 0 (SWC: men 85 cm and women 90 cm) and 25 (BMI). Each cell represents a coefficient with 95% confidence interval from a separate regression. In all regressions, we used a triangular kernel function, which assigns more weight to observations closer to the thresholds. The models in the second column do not include any covariates; those in the third column include demographic covariates (age and sex); those in the fourth column additionally include socioeconomic covariates (income and occupation status); and those in the fifth column additionally include behavioural covariates (smoking, alcohol drinking, and regular exercise). Baseline waist circumference and BMI were used as assignment variables.

†Δ represents the change of the measurements.

For the estimates of local quadratic linear regression shown in Table S1 in the **Online Supplementary Document**, similar results were observed based on health checkups using waist circumference, although the change in waist circumference was marginally reduced by the intervention. No observed significant reductions in obesity, cardiovascular, and health care utilisation outcomes were observed based on health checkups using waist circumference, except for a reduction in diastolic blood pressure. Estimates according to sex and age presented comparable results (Tables S2 and S3 in the **Online Supplementary Document**). A significant reduction in obesity outcomes was observed in women, and a slight reduction in obesity was observed in men, based on health checkups using waist circumference. A decrease in inpatient costs was also observed in men, based on health checkups using BMI. Additionally, a significant reduction in weight and BMI was observed among the age groups 40–54 and 55–64 years based on health checkups using waist circumference, and outpatient costs were significantly reduced among those in the 65–74-year age group.

**Figure 3:**
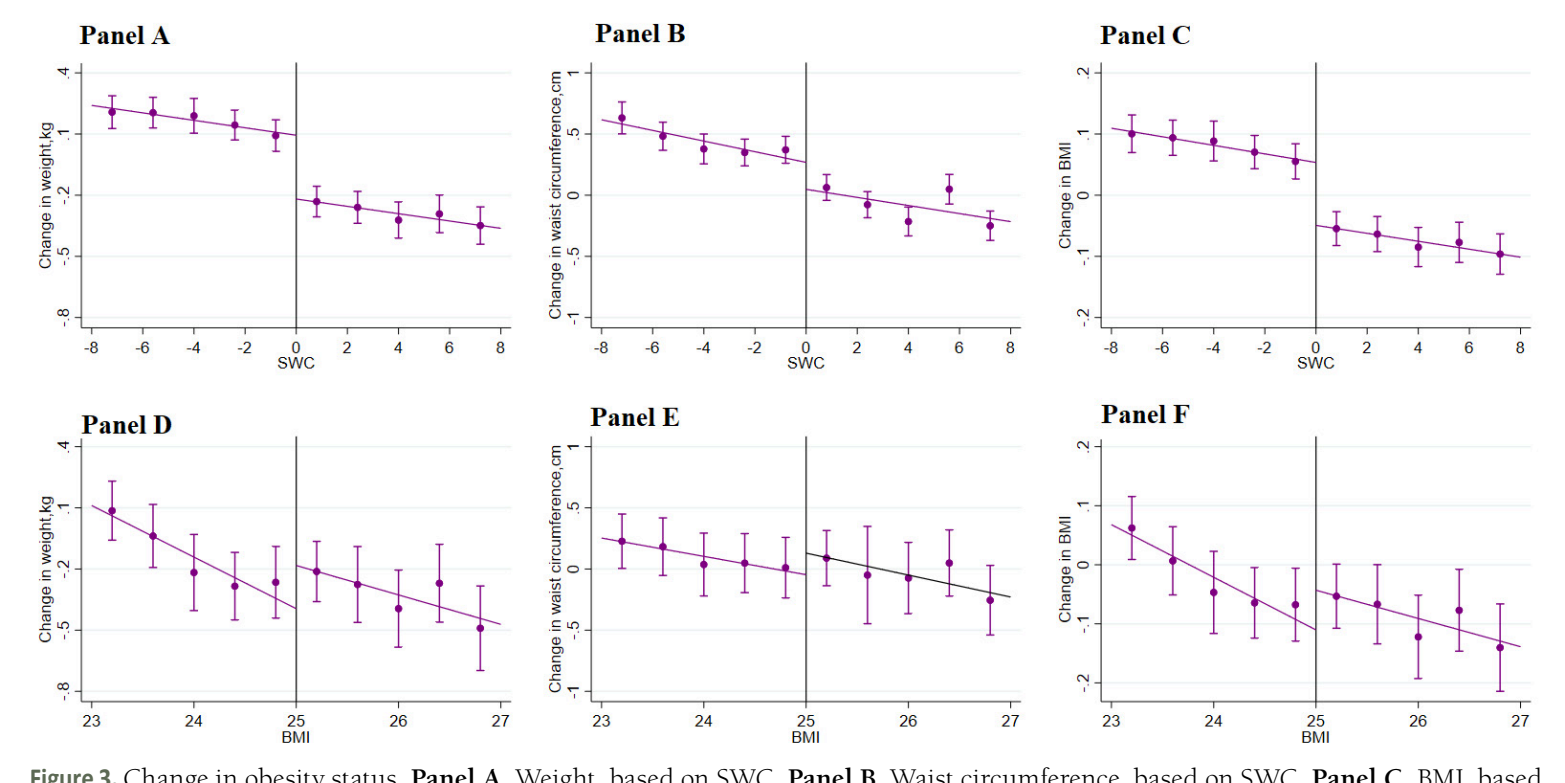
Change in obesity status. **Panel A.** Weight, based on SWC. **Panel B.** Waist circumference, based on SWC. **Panel C.** BMI, based on SWC. **Panel D.** Weight, based on BMI. **Panel E.** Waist circumference, based on BMI. **Panel F.** BMI, based on BMI. Changes in obesity status (weight, WC, and BMI) in 2016 across the range of changes in obesity outcomes in 2015 within the selected bandwidths. The vertical dashed lines represent the thresholds of the assignment variables (SWC or BMI); the dots and error bars represent the point estimates and 95% confidence intervals. The total sample size was 46 975, and the sample size within the selected optimal bandwidth was 28 046 for WC and 6110 for BMI. BMI – body mass index, SWC – standardised waist circumference

**Figure 4:**
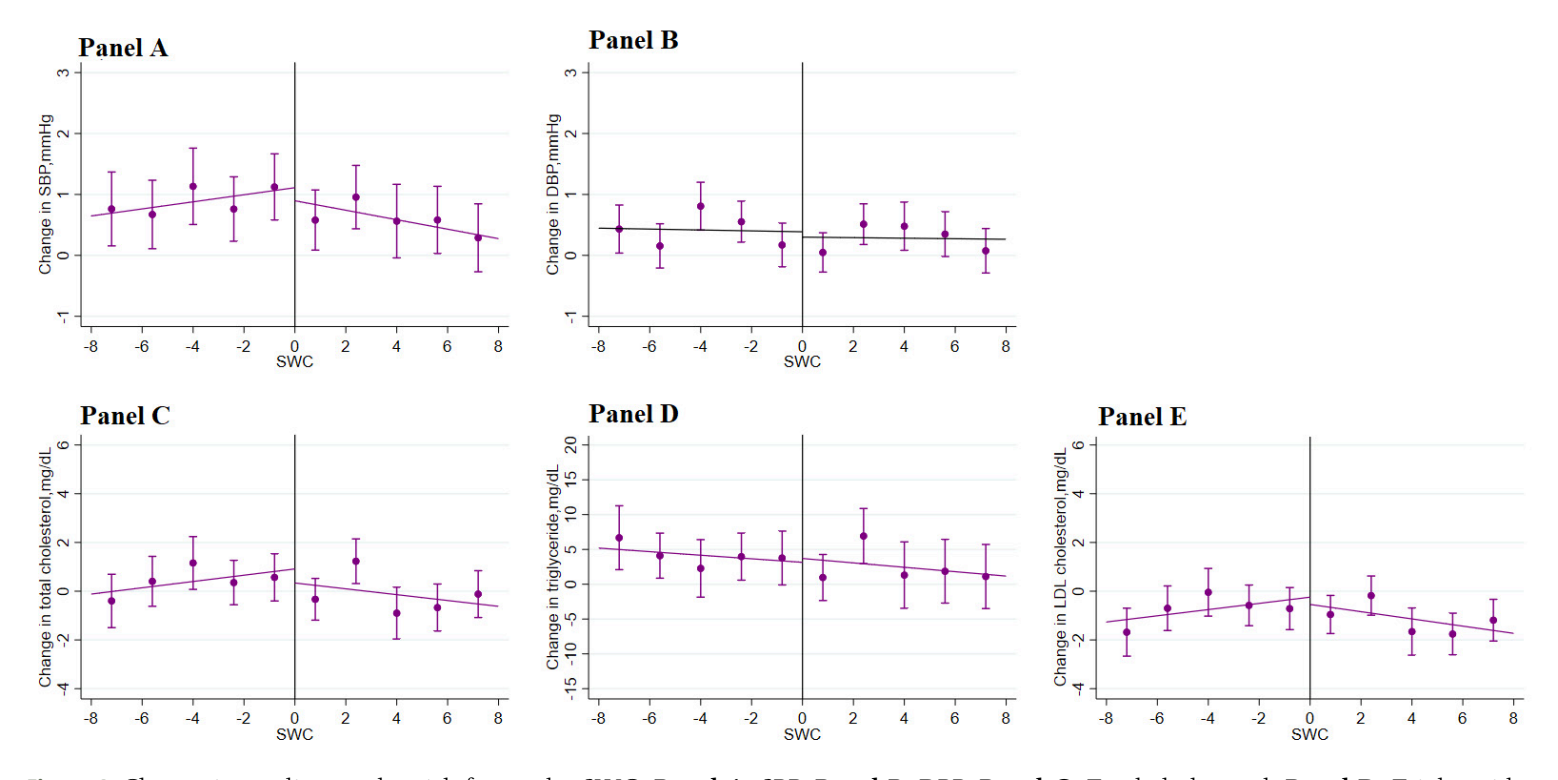
Change in cardiovascular risk factors by SWC. **Panel A.** SBP. **Panel B.** DBP. **Panel C.** Total cholesterol. **Panel D.** Triglyceride. **Panel E.** LDL cholesterol. Changes in cardiovascular risk factors in 2016 across the range of changes in obesity outcomes in 2015 within the selected bandwidths. The vertical dashed lines represent the thresholds of the assignment variables; the dots and error bars represent the point estimates and 95% confidence intervals. The total sample size was 46 975; the sample size within the selected optimal bandwidth was 28 046. BMI – body mass index, DBP – diastolic blood pressure, LDL – low-density lipoprotein, SBP – systolic blood pressure, SWC – standardised waist circumference

**Figure 5:**
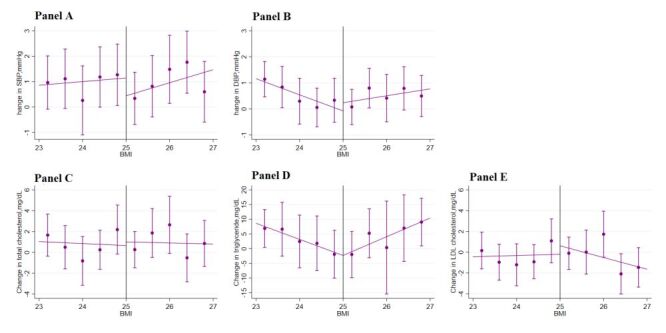
Change in cardiovascular risk factors by BMI. **Panel A.** SBP. **Panel B.** DBP. **Panel C**. Total cholesterol. **Panel D.** Triglyceride. **Panel E.** LDL cholesterol. Changes in cardiovascular risk factors in 2016 across the range of changes in obesity outcomes in 2015 within the selected bandwidths. The vertical dashed lines represent the thresholds of the assignment variables; the dots and error bars represent the point estimates and 95% confidence intervals. The total sample size was 46 975; the sample size within the selected optimal bandwidth was 6110. BMI – body mass index, DBP – diastolic blood pressure, LDL – low-density lipoprotein, SBP – systolic blood pressure, SWC – standardised waist circumference

**Figure 6:**
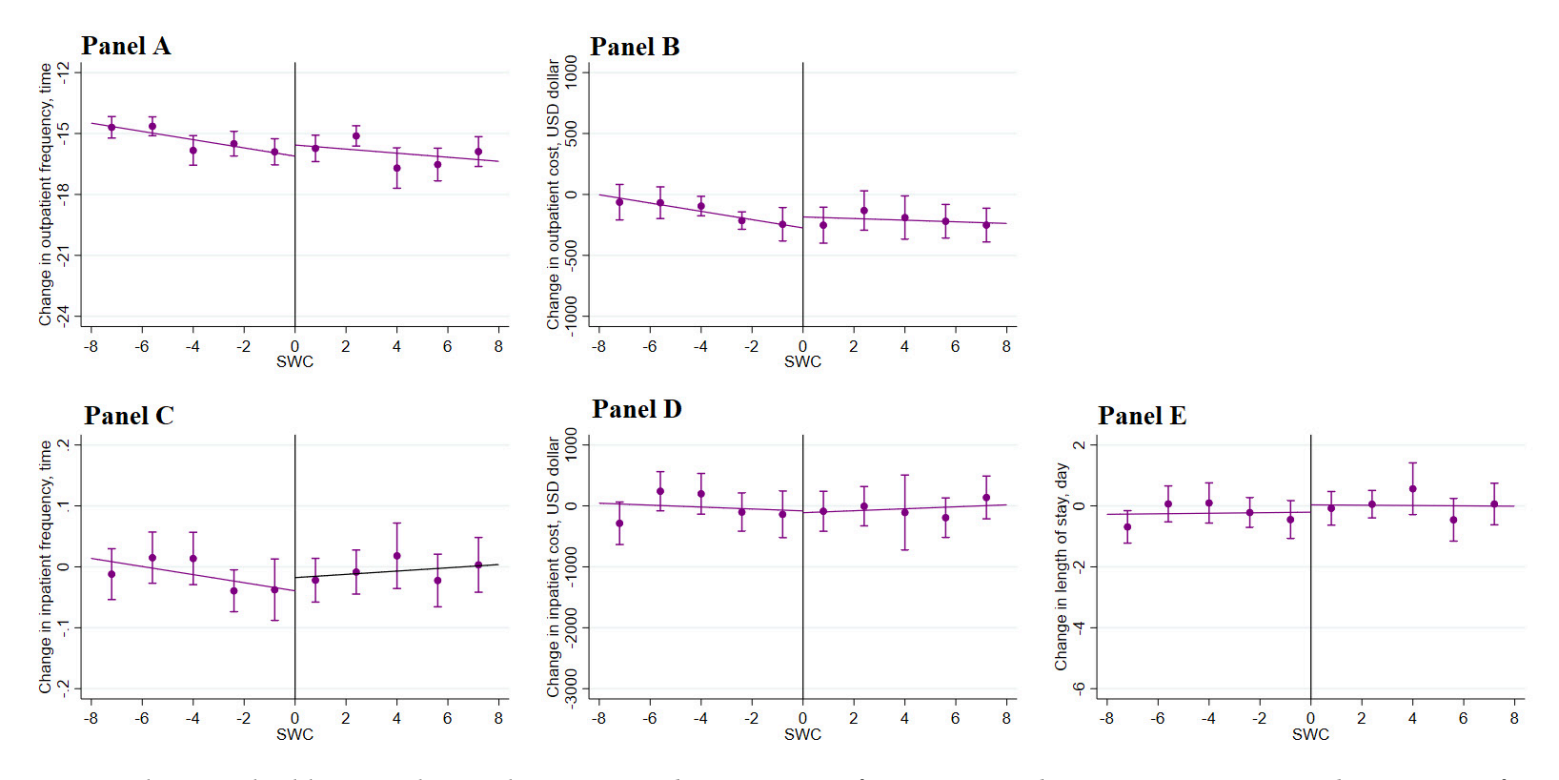
Change in health care utilisation by SWC. **Panel A.** Outpatient frequency. **Panel B.** Outpatient cost. **Panel C.** Inpatient frequency. **Panel D.** Inpatient cost. **Panel E.** Length of stay. Change in health care utilisation in 2016 across the range of changes in obesity outcomes in 2015 within the selected bandwidths. The vertical dashed lines represent the thresholds of the assignment variables; the dots and error bars represent the point estimates and 95% confidence intervals. The total sample size was 46 975; the sample size within the selected optimal bandwidth was 28 046. BMI – body mass index, SWC – standardised waist circumference

**Figure 7:**
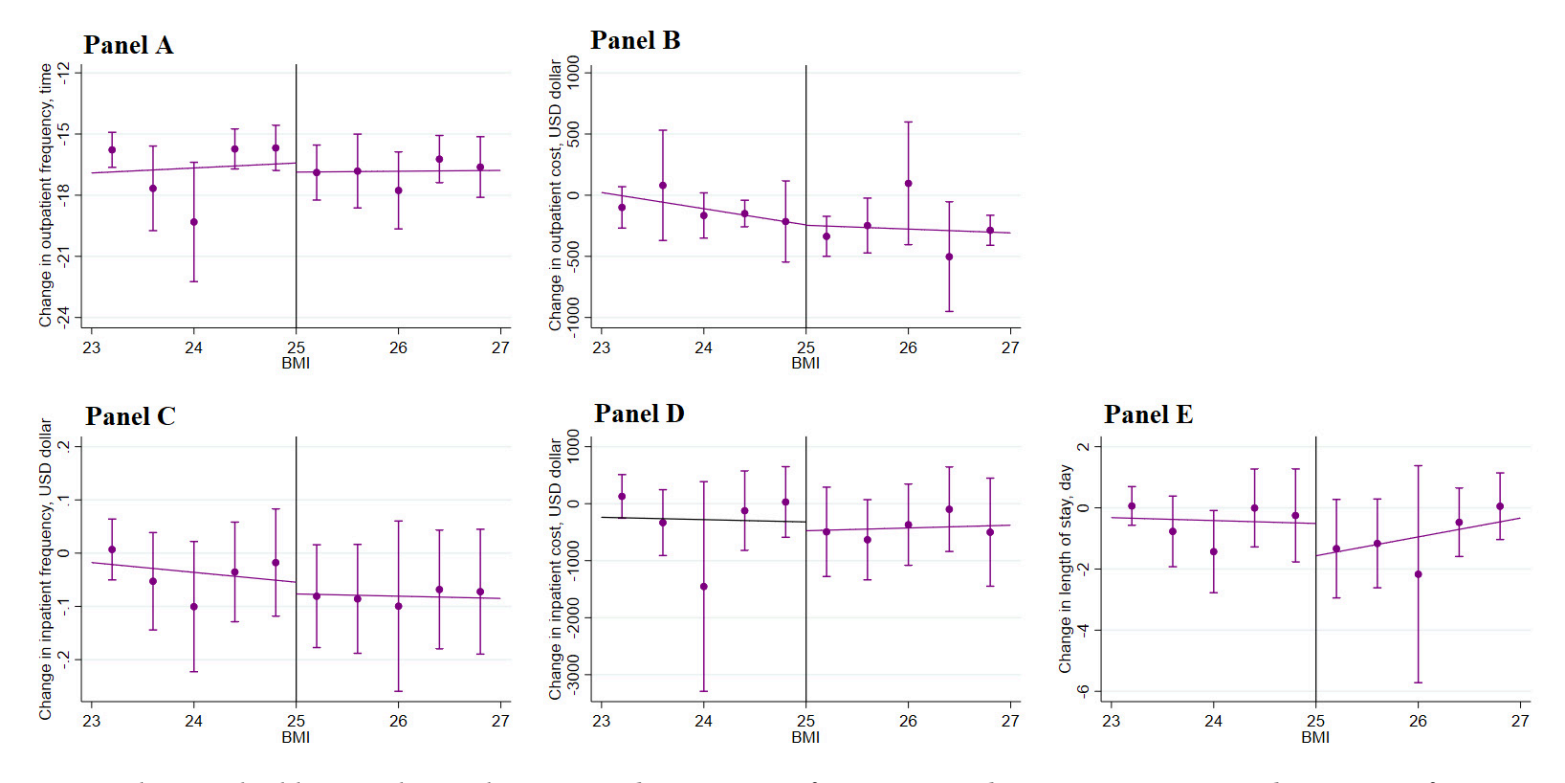
Change in health care utilisation by BMI. **Panel A.** Outpatient frequency. **Panel B.** Outpatient cost. **Panel C.** Inpatient frequency. **Panel D.** Inpatient cost. **Panel E.** Length of stay. Change in health care utilisation in 2016 across the range of changes in obesity outcomes in 2015 within the selected bandwidths. The vertical dashed lines represent the thresholds of the assignment variables; the dots and error bars represent the point estimates and 95% confidence intervals. The total sample size was 46 975; the sample size within the selected optimal bandwidth was 6110. BMI – body mass index, SWC – standardised waist circumference

**Figure 3, **panels A–F displays the main outcomes of the study, including change in obesity (change in weight, waist circumference, and BMI) within one year across a range of assignment variables (SWC and BMI in 2015) within the selected optimal bandwidths. We observed a sharp downward change in weight, waist circumference, and BMI across the discontinuity in SWC. This graphical presentation indicates a positive impact of the lifestyle guidance intervention in 2015 on the change in obesity outcomes in 2016 based on the criteria of waist circumference. In contrast, we failed to observe a downward change in these obesity outcomes when the intervention selection was based on BMI. Likewise, we did not observe a meaningful change in other risk factors (**Figure 4, **panels A–E and** Figure 5, **panels A–E) or health care utilisation outcomes across the discontinuity in either SWC (**Figure 6, **panels A–E) or BMI (**Figure 7**, panels A–E).

### Changes in health behaviour

The estimates of the changes in health behaviours are presented in Table S4 in the **Online Supplementary Document**. The results were similar for individuals who received interventions based on SWC and BMI. We found evidence that lifestyle guidance interventions could lead to improvements in dietary habits. In contrast, lifestyle guidance interventions were less likely to yield a positive behavioural change for smoking, drinking, regular exercise, and sleeping habits. This estimation of changes in health behaviours provides limited support for the positive impact of lifestyle guidance interventions in general. However, it is important to acknowledge that we lacked the data needed to estimate some potentially important changes, such as the daily consumption of carbohydrates, protein, vegetables, and fruits.

### Robustness checks

We tested the robustness of our findings by examining the influence of impact estimates over multiple bandwidths on SWC and BMI. We used bandwidths of 50, 80, 120, and 150% of the optimal bandwidth (100%) to observe the variation in the results (Table S5 in the **Online Supplementary Document**). Statistically, the estimates did not show a remarkable change resulting from the change in bandwidth for either threshold (SWC and BMI). The robustness checks further supported the design and the results of the estimation regarding the impact of lifestyle guidance intervention for adults with metabolic syndrome on health outcomes.

## DISCUSSION

### Lifestyle guidance intervention based on waist circumference

In the primary analyses, a statistically significant but rather small reduction in obesity (reduction in waist circumference ranged from 0.02 to 0.5 cm; reduction in weight ranged from 0.1 to 0.5 kg) was observed among participants selected for intervention based on waist circumference. These reductions were far less than the recommendations of some clinical guidelines for the management of overweight and obesity (3 kg weight loss or 3 cm waist circumference reduction) [30,31]. Additionally, no significant decrease was observed in cardiovascular risk factors and health care utilisation, which may be owing to the relatively low cutoff point of the age-specified waist circumference (85 cm for men and 90 cm for women) applied for the diagnosis of metabolic syndrome in Japan [32]. Another possible reason is that all participants, including those who met the criteria for a diagnosis of metabolic syndrome, would receive messages notifying them of their health checkup results together with healthy lifestyle promotional materials, which could have influenced individuals to adopt healthy behaviours, even without receiving a formal intervention. Further analyses on the impact of the behavioural change showed that the lifestyle guidance intervention only resulted in changing poor dietary habits, and the expected benefits of this intervention in changing smoking and alcohol consumption behaviours, as well as in adopting regular physical activity and healthy sleeping patterns, were not observed. A possible explanation for these findings is that lifestyle guidance interventions mainly focus on self-health management, with little emphasis on behaviour reinforcement. Overall, our analyses provide evidence for the need to improve this interventional programme in the future.

### Lifestyle guidance intervention based on BMI

Lifestyle guidance intervention based on BMI had no impact on the reduction of any health outcomes and health care utilisation. One possible reason for this finding is that individuals who were enrolled in this intervention based on their BMI had at least two cardiovascular risk factors; therefore, their lifestyles and health conditions would be more difficult to change or improve through self-management alone. Additionally, despite the use of BMI as a gold standard in categorising body weight ranges [33], the applicability of BMI in diagnosing obesity in adult populations has been consistently challenged because it fails to consider sex and age variations among adults [34,35]. Instead, the use of waist circumference provides a more reliable criterion for enrollment in lifestyle guidance interventions because it is also considered an important indicator of cardiometabolic morbidity and mortality [36]. Whether BMI should continue to be used as a criterion for receiving lifestyle intervention in this national programme warrants further discussion.

### Comparison with published evidence

Our results suggest that the improvement in health outcomes and health care utilisation resulting from the national lifestyle guidance intervention programme in Japan is limited. However, our findings were consistent with previous studies which demonstrated that weight loss was minimally or even inversely associated with lifestyle interventions for metabolic syndrome [13,37]. In contrast, positive effects of lifestyle interventions on weight loss, cardiovascular diseases, and health care utilisation have also been found in Korea [38], Australia [39], Italy [40], and the United Kingdom [41], which involved more intensive clinical intervention methods. Hence, our results suggest the need to re-evaluate the existing diagnostic criteria and form a more engaging lifestyle intervention for metabolic syndrome in Japan.

### Health policy implications

Our results suggest that the delivery of lifestyle guidance interventions based on national health checkups should be further improved. Evidence from Japan and other Asian countries shows that there is a great need to enhance awareness of lifestyle behavioural changes among adults with metabolic syndrome. Although interventions can provide opportunities for at-risk populations to gain access to knowledge about metabolic syndrome, findings regarding the efficacy of some potentially promising interventions from large-scale clinical and epidemiological evidence based on Asian populations are needed. Additionally, because the current intervention programme overemphasises improvements in individual awareness and self-management of health conditions, the use of other evidence-based intervention methods, designs, and publicity campaign activities should also be considered. An intensive internet-based lifestyle intervention that monitors and alerts participants regarding their daily dietary intake and physical activity can be incorporated into this national programme [10,41]. Overall, to optimise the impact of this expansive national intervention programme, policymakers and experts should work together to reevaluate the diagnostic criteria of metabolic syndrome and to develop the content of the intervention.

### Limitations

This study has some limitations. First, the existing diagnostic criteria for metabolic syndrome in Japan are determined according to their unique population characteristics, culture, and dietary habits; thus, experts and policymakers in other countries should consider these factors and specify their diagnostic criteria and guidelines. Second, the outcomes might be affected by the varied duration of follow-up. Given that some participants may receive health checkups more than once per year, only the first checkup was analysed, resulting in a varying follow-up period ranging from one month to two years rather than the strict follow-up period in this intervention programme (three to six months). However, the main purpose for this study is to evaluate whether the national intervention can improve the health outcomes as a result of increased public awareness of metabolic syndrome and its associated risk factors; therefore, the influence of education and counselling following the health checkup deserves more attention than the variability of follow-up duration. Third, a few studies reported that the follow-up implementation rates for screened hypertension and hyperglycemia were low (10–35%) [42,43], considering the number of follow-up consultations and those who finished the whole intervention programme (three to six months). Most of the eligible people attended the initial interview soon after the health checkups; however, the high dropout rates could not be ignored and require further investigation. Fourth, this study only obtained data from 2015 and 2016; therefore, further studies with longer follow-up periods are required. Despite these limitations, we successfully linked national health checkup data and health care claims data, with coverage of more than 350 000 population which obtained statistically robust findings.

## CONCLUSIONS

Lifestyle guidance intervention had limited effects on both health outcomes and health care utilisation among adults with metabolic syndrome at the population level, which may be attributed to stringent diagnostic criteria for metabolic syndrome in Japan. Policymakers should reevaluate the current diagnostic criteria for participation and the content of the lifestyle guidance intervention. The exploration of other strategies that could improve health outcomes and reduce health care utilisation among the adult population is expected to maximise the benefits of this nationwide programme.

## Additional material


Online Supplementary Document
