# Supplementary material for: Effect of the national lifestyle guidance intervention for metabolic syndrome among middle-aged people in Japan
Source: J Glob Health. 2024 Feb 9;14:04007. doi: 10.7189/jogh.14.04007 (PMC10854208; doi:10.7189/jogh.14.04007)
Supplement: Online Supplementary Document [file jogh-14-04007-s001.pdf]

SUPPLEMENTARY DOCUMENT

Box 1. National health checkups and lifestyle guidance intervention program in Japan

1. Health checkups

Health checkups are held annually for individuals aged 40–74 years. Items in the health checkups mainly include questionnaires and physical examinations. Questionnaires are focused on current health conditions and lifestyle behaviors, including history of disease, medications currently taken, current smoking, alcohol drinking, exercise habits, dietary habits, and sleep hygiene. Physical examinations include measurement of waist circumference, weight, body mass index (BMI), and blood pressure as well as blood tests and urinalysis. All exams are conducted under an ordinance of the Ministry of Health, Labour and Welfare. Health checkup results are provided to all participants in the form of a notification, regardless of the presence or absence of metabolic syndrome.

2. Lifestyle guidance intervention

2.1. Selection criteria for lifestyle guidance intervention

The judgment of whether a participant needs to receive lifestyle guidance intervention is made by a physician based on the following health checkup items. These items are a participant's: obesity status (waist circumference and BMI) and cardiovascular status (hypertension, diabetes, and dyslipidemia). The selection of participants who should receive lifestyle guidance intervention mainly follows a two-stage process, as shown as Figure S1.

Stage 1: Selection based on waist circumference

The primary selection criterion is the participant's waist circumference. If waist circumference is more than the cutoff (men ≥85 cm and women ≥90 cm), a physician will estimate the participant's health condition based on their cardiovascular risk factors. If the participant has more than one cardiovascular risk factor, they will be considered to have metabolic syndrome and will require lifestyle guidance intervention. Based on the number of cardiovascular risk factors, different types of lifestyle guidance intervention will be provided, namely, motivational support or intensive support. Motivational support is provided if the participant has only one cardiovascular risk factor; intensive support is provided if the participant has two or more cardiovascular risk factors. The lifestyle guidance intervention will not be provided to participants with no cardiovascular risk factors. The detailed content of motivational support and intensive support is discussed in the next section.

Stage 2: Selection based on BMI

The secondary selection criterion is the participant's BMI. If BMI is more than 25 kg/m^2, a physician will estimate the participant's health condition based on their cardiovascular risk factors to decide the type of lifestyle guidance intervention to be provided. Motivational support will be provided if the participant has two cardiovascular risk factors; intensive support will be provided if the participant has more than two cardiovascular risk factors. Lifestyle guidance intervention will not be provided if the participant has one or no cardiovascular risk factors.

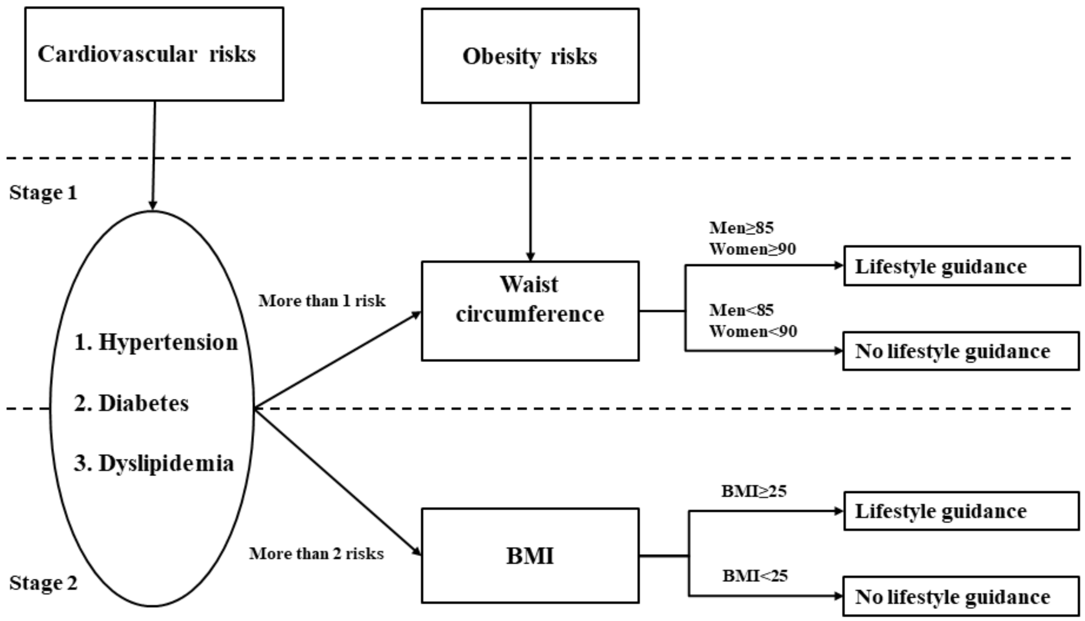

Figure S1. Criteria of metabolic syndrome and flow of lifestyle guidance intervention for adults with metabolic syndrome in Japan. Cardiovascular risk factors include (1) hypertension: systolic blood pressure ≥130 mm Hg or diastolic blood pressure ≥85 mm Hg; (2) diabetes: fasting blood glucose ≥100 mg/dL or hemoglobin A1c ≥5.6%; and (3) dyslipidemia: triglyceride ≥150 mg/dL or high-density lipoprotein cholesterol <40 mg/dL.

2.2. Lifestyle guidance intervention

Participants who receive the lifestyle guidance intervention are allocated into either a moderate support group or intensive support group according to their obesity status (waist circumference/body mass index [BMI]) and cardiovascular risk factors, as described above. Participants receive an initial interview and an evaluation after 3 months or an additional final evaluation after 6 months depending on the participant’s achievement of goals, as shown in Figure S2.

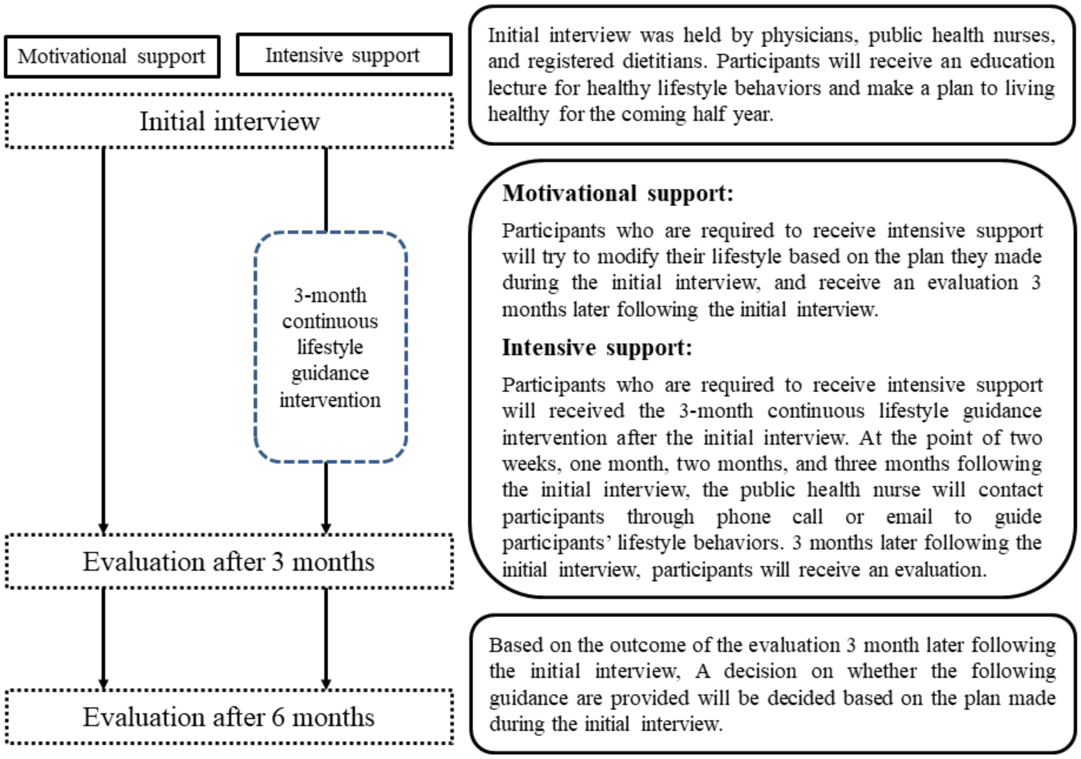

Figure S2. Type and content of lifestyle guidance intervention

2.2.1. Motivational support

Participants who are required to receive motivational support attend an initial interview, which includes an individual interview and group interview. Individual interviews last for 20 minutes or more per participant, and group interviews are 80 minutes or more per group. During the interview, participants receive an educational lecture regarding healthy lifestyle behaviors and set half-year goals together with physicians, public health nurses, and registered dietitians. Participants try to modify their health behaviors according to the guidance given during the interview and undergo an evaluation 3 months after the initial interview. Whether to perform a 6-month lifestyle guidance intervention is decided based on the achievement of goals.

2.2.2. *Intensive support*

Participants who are required to receive intensive support attend an initial interview, which includes an individual interview and group interview. Individual interviews are 20 minutes or more per participant, and group interviews are 80 minutes or more per group. During the interview, participants receive an educational lecture regarding healthy lifestyle behaviors and set half-year goals together with physicians, public health nurses, and registered dietitians. Participants receive guidance on their progress toward goals from public health nurses by telephone or email at 2 weeks, 1 month, 2 months, and 3 months following the initial interview. At 3 months, participants undergo an evaluation to assess their progress and outcomes. Whether to perform 6-month lifestyle guidance intervention is decided based on the achievement of goals.

2.2.3. *No lifestyle guidance intervention*

Participants who do not require lifestyle guidance intervention receive a summary report by mail or email to help increase participants' awareness of their health condition and consider reviewing their lifestyle choices.

Table S1. Regression discontinuity estimates (95% confidence intervals) of the impact of lifestyle guidance intervention in 2015 on health outcomes in 2016, fitted using local quadratic regression

	SWC				BMI			
Impact of lifestyle guidance intervention	Without covariates	With demographic covariates	With demographic and socioeconomic covariates	With demographic, socioeconomic, and behavioral covariates	Without covariates	With demographic covariates	With demographic and socioeconomic covariates	With demographic, socioeconomic, and behavioral covariates
**Obesity outcomes**								
Δ Weight	–0.30 (–0.51 to –0.08, *P*=0.007)	–0.31 (–0.50 to –0.08, *P*=0.008)	–0.31 (–0.50 to –0.08, *P*=0.008)	–0.29 (–0.52 to –0.05, *P*=0.020)	0.07 (–0.45 to 0.56, *P*=0.837)	0.08 (–0.45 to 0.56, *P*=0.833)	0.07 (–0.45 to 0.56, *P*=0.837)	0.14 (–0.44 to 0.74, *P*=0.619)
Δ waist circumference	–0.31 (–0.63 to 0.01, *P*=0.053)	–0.29 (–0.61 to 0.02, *P*=0.064)	–0.29 (–0.61 to 0.02, *P*=0.063)	–0.28 (–0.64 to 0.05, *P*=0.092)	0.13 (–0.63 to 0.78, *P*=0.828)	0.14 (–0.62 to 0.78, *P*=0.817)	0.13 (–0.62 to 0.78, *P*=0.820)	0.01 (–0.91 to 0.69, *P*=0.791)
ΔBMI	–0.11 (–0.18 to –0.02, P=0.011)	–0.11 (–0.18 to –0.02, P=0.011)	–0.11 (–0.18 to –0.02, P=0.011)	–0.11 (–0.19 to –0.02, P=0.022)	0.03 (–0.15 to 0.21, *P*=0.716)	0.03 (–0.15 to 0.21, *P*=0.712)	0.03 (–0.15 to 0.21, *P*=0.716)	0.05 (–0.14 to 0.28, *P*=0.528)
**Cardiovascular outcomes**								
Δ Systolic blood pressure	–0.28 (–1.63 to 1.30, *P*=0.826)	–0.28 (–1.63 to 1.31, *P*=0.832)	–0.28 (–1.63 to 1.31, *P*=0.830)	–0.57 (–1.92 to 1.28, *P*=0.699)	–1.04 (–3.86 to 3.25, *P*=0.866)	–1.04 (–3.86 to 3.25, *P*=0.868)	–1.04 (–3.86 to 3.25, *P*=0.867)	–2.22 (–6.32 to 1.49, *P*=0.226)
Δ Diastolic blood pressure	0.07 (–0.56 to 1.33, *P*=0.422)	0.08 (–0.55 to 1.34, *P*=0.411)	0.08 (–0.55 to 1.34, *P*=0.416)	–0.06 (–0.67 to 1.39, *P*=0.499)	–0.82 (–3.89 to 0.87, *P*=0.214)	–0.82 (–3.89 to 0.87, *P*=0.214)	–0.83 (–3.89 to 0.87, *P*=0.213)	–2.08 (–5.94 to –0.69, *P*=0.013)
Δ Total cholesterol	–0.68 (–4.51 to 0.86, *P*=0.183)	–0.70 (–4.52 to 0.85, *P*=0.179)	–0.71 (–4.55 to 0.82, *P*=0.174)	–1.13 (–5.24 to 0.57, *P*=0.115)	–0.68 (–4.51 to 0.86, *P*=0.183)	–0.70 (–4.52 to 0.85, *P*=0.179)	–0.71 (–4.55 to 0.82, *P*=0.174)	–1.13 (–5.24 to 0.57, *P*=0.115)
Δ Triglyceride	–2.22 (–8.42 to 11.12, *P*=0.787)	–2.18 (–8.37 to 11.15, *P*=0.781)	–2.21 (–8.42 to 11.11, *P*=0.787)	0.01 (–6.93 to 14.89, *P*=0.475)	–2.22 (–8.42 to 11.12, *P*=0.787)	–2.18 (–8.37 to 11.15, *P*=0.781)	–2.21 (–8.42 to 11.11, *P*=0.787)	0.01 (–6.93 to 14.89, *P*=0.475)
ΔLDL cholesterol	0.05 (–3.23 to 1.52, *P*=0.482)	0.04 (–3.24 to 1.51, *P*=0.475)	0.03 (–3.25 to 1.49, *P*=0.467)	–0.64 (–4.23 to 0.97, *P*=0.218)	–2.56 (–7.89 to 2.82, *P*=0.354)	–2.61 (–7.92 to 2.79, *P*=0.348)	–2.58 (–7.90 to 2.80, *P*=0.349)	–1.99 (–8.56 to 3.25, *P*=0.378)
**Healthcare utilization outcomes**								
Δ Outpatient frequency	0.05 (–2.39 to 1.51, *P*=0.660)	–0.06 (–2.45 to 1.42, *P*=0.604)	–0.02 (–2.38 to 1.48, *P*=0.645)	0.26 (–2.16 to 2.18, *P*=0.991)	–2.27 (–4.23 to 2.51, *P*=0.617)	–2.29 (–4.25 to 2.49, *P*=0.609)	–2.37 (–4.26 to 2.46, *P*=0.599)	–2.10 (–4.41 to 3.38, *P*=0.796)
Δ Outpatient cost	95.6 (–253.1 to 402.7, *P*=0.655)	91.5 (–256.1 to 399.5, *P*=0.668)	93.1 (–253.7 to 401.7, *P*=0.658)	78.7 (–323.5 to 428.7, *P*=0.784)	–274.5 (–836.5 to 114.7, *P*=0.137)	–276.1 (–837.3 to 113.2, *P*=0.135)	–273.1 (–836.1 to 114.1, *P*=0.136)	–314.6 (–968.2 to 170.5, *P*=0.170)
Δ Inpatient frequency	0.01 (–0.15 to 0.07, *P*=0.482)	0.01 (–0.15 to 0.07, *P*=0.475)	0.01 (–0.15 to 0.07, *P*=0.472)	–0.01 (–0.17 to 0.09, *P*=0.545)	0.01 (–0.15 to 0.07, *P*=0.482)	0.01 (–0.15 to 0.07, *P*=0.475)	0.01 (–0.15 to 0.07, *P*=0.472)	–0.01 (–0.17 to 0.09, *P*=0.545)
Δ Inpatient cost	183.9 (–1130.1 to 407.1, *P*=0.419)	180.9 (–1133.1 to 467.3, *P*=0.415)	178.7 (–1136.7 to 463.4, *P*=0.410)	127.6 (–1253.8 to 503.3, *P*=0.403)	–1414.2 (–3054.0 to 625.6, *P*=0.196)	–1429.4 (–3066.8 to 616.6, *P*=0.192)	–1430.6 (–3066.6 to 616.4, *P*=0.196)	–1795.2 (–3725.0 to 720.7, *P*=0.185)
Δ Length of stay	0.49 (–1.46 to 1.14, *P*=0.809)	0.49 (–1.47 to 1.14, *P*=0.801)	0.48 (–1.48 to 1.13, *P*=0.798)	0.26 (–1.75 to 1.19, *P*=0.708)	–0.90 (–5.06 to 5.24, *P*=0.974)	–0.91 (–5.08 to 5.23, *P*=0.977)	–0.93 (–5.09 to 5.22, *P*=0.980)	–1.09 (–5.88 to 6.80, *P*=0.887)

The sample comprises people within optimal bandwidth of the threshold 0 (SWC: men 85 cm and women 90 cm) and 25 kg/m^2 (BMI). Each cell represents the coefficient with 95% confidence interval from a separate regression. In all regressions, we used a triangular kernel function, which gives more weight to observations closer to the thresholds. Models in the second column do not include any covariates; models in the third column include demographic covariates (age and sex); models in the fourth column additionally include socioeconomic covariates (income and occupation); and models in the fifth column additionally include behavioral covariates (smoking, alcohol drinking, and regular exercise). Baseline waist circumference and BMI are used as assignment variables.

Abbreviations: SWC, standardized waist circumference; BMI, body mass index; LDL, low-density lipoprotein.

Table S2. Regression discontinuity estimates (95% confidence intervals) of the impact of lifestyle guidance intervention in 2015 on outcome changes in 2016 by sex, fitted using local linear regression

	SWC		BMI	
Impact of lifestyle guidance intervention	Men	Women	Men	Women
**Obesity outcomes**				
Δ Weight	–0.21 (–0.37 to 0.03, *P*=0.093)	–0.52 (–1.11 to –0.33, *P*<0.001)	0.07 (–0.48 to 0.48, *P*=0.997)	0.49 (–0.10 to 1.30, *P*=0.093)
Δ WC	–0.21 (–0.45 to 0.09, *P*=0.192)	–0.45 (–1.27 to –0.03, *P*=0.041)	–0.07 (–0.62 to 0.62, *P*=0.999)	0.47 (–1.33 to 1.43, *P*=0.943)
ΔBMI	–0.07 (–0.13 to –0.01, *P*=0.05)	–0.19 (–0.44 to –0.12, *P*=0.001)	0.02 (–0.17 to 0.16, *P*=0.953)	0.16 (–0.06 to 0.52, *P*=0.124)
**Cardiovascular outcomes**				
Δ Systolic blood pressure	0.07 (–1.00 to 1.46, *P*=0.901)	–1.39 (–5.40 to –0.17, *P*=0.037)	–1.22 (–4.08 to 1.31, *P*=0.247)	–3.16 (–9.47 to 2.23, *P*=0.225)
Δ Diastolic blood pressure	–0.14 (–0.85 to 1.08, *P*=0.977)	–0.16 (–1.98 to 1.42, *P*=0.746)	–1.11 (–0.62 to 0.62, *P*=0.010)	–0.07 (–0.62 to 0.62, *P*=0.999)
Δ Total cholesterol	–0.88 (–3.75 to 0.75, *P*=0.191)	–0.94 (–5.30 to 5.15, *P*=0.978)	1.19 (–6.11 to 4.76, *P*=0.808)	–5.51 (–20.41 to 0.92, *P*=0.073)
Δ Triglyceride	1.45 (–10.38 to 9.47, *P*=0.928)	1.34 (–10.01 to 14.03, *P*=0.743)	2.91 (–24.22 to 31.57, *P*=0.796)	–2.42 (–26.44 to 12.46, *P*=0.481)
ΔLDL cholesterol	–0.56 (–3.01 to 1.05, *P*=0.344)	–0.85 (–4.48 to 4.84, *P*=0.940)	0.74 (–0.62 to 0.62, *P*=0.695)	–0.07 (–5.76 to 3.84, *P*=0.219)
**Healthcare utilization outcomes**				
Δ Outpatient frequency	0.58 (–1.75 to 2.24, *P*=0.809)	0.91 (–0.91 to 2.26, *P*=0.403)	–1.23 (–5.68 to 1.39, *P*=0.235)	–0.50 (–7.18 to 3.52, *P*=0.502)
Δ Outpatient cost	96.9 (–199.3 to 471.7, *P*=0.426)	–10.2 (–436.2 to 178.9, *P*=0.412)	34.7 (–982.9 to 403.4, *P*=0.413)	–2823.0 (–970.1 to 112.8, *P*=0.121)
Δ Inpatient frequency	–0.02 (–0.13 to 0.10, *P*=0.803)	0.05 (–0.09 to 0.16, *P*=0.581)	–0.05 (–0.41 to 0.14, *P*=0.340)	–0.02 (–0.35 to 0.46, *P*=0.787)
Δ Inpatient cost	–129.2 (–800.2 to 837.7, *P*=0.964)	469.2 (–438.1 to 1582.3, *P*=0.267)	–848.3 (–4447.4 to 198.4, *P*=0.032)	–496.5 (–1333.6 to 1756.8, *P*=0.788)
Δ Length of stay	–0.12 (–1.19 to 1.49, *P*=0.824)	0.73 (–1.01 to 2.59, *P*=0.824)	–2.14 (–6.99 to 0.89, *P*=0.129)	2.25 (–7.66 to 20.02, *P*=0.381)

The sample comprises people within optimal bandwidth of the threshold 0 (SWC: men 85 cm and women 90 cm) and 25 kg/m^2 (BMI). Each cell represents the coefficient with 95% confidence interval from a separate regression. In all regressions, we used a triangular kernel function, which gives more weight to observations closer to the thresholds. All models in these analyses include demographic (age and sex), socioeconomic (income and occupation), and behavioral covariates (smoking, alcohol drinking, regular exercise, and dietary habits). Baseline SWC and BMI in 2015 are used as assignment variables. Abbreviations: SWC, standardized waist circumference; BMI, body mass index; LDL, low-density lipoprotein.

Table S3. Regression discontinuity estimates (95% confidence intervals) of the impact of lifestyle guidance intervention in 2015 on outcome changes in 2016 by age categories, fitted using local linear regression

	SWC			BMI		
Impact of lifestyle guidance intervention	40–54	55–64	65–74	40–54	55–64	65–74
**Obesity outcomes**						
Δ Weight	–0.28 (–0.72 to –0.01, *P*=0.045)	–0.28 (–0.52 to –0.03, *P*=0.029)	–0.29 (–0.53 to –0.10, *P*=0.186)	–0.18 (–1.62 to 0.24, *P*=0.146)	0.21 (–0.16 to 0.91, *P*=0.173)	0.36 (–0.46 to 1.17, *P*=0.394)
Δ WC	–0.25 (–0.85 to 0.09, *P*=0.111)	–0.22 (–0.56 to 0.17, *P*=0.295)	–0.40 (–0.87 to 0.15, *P*=0.165)	–0.19 (–2.00 to 0.45, *P*=0.214)	0.23 (–0.39 to 1.12, *P*=0.340)	–0.20 (–1.24 to 1.12, *P*=0.917)
ΔBMI	–0.09 (–0.26 to 0.003, *P*=0.044)	–0.10 (–0.19 to 0.01, *P*=0.032)	–0.09 (–0.20 to 0.04, *P*=0.204)	–0.06 (–0.53 to 0.11, *P*=0.204)	–0.07 (–0.08 to 0.30, *P*=0.253)	0.11 (–0.17 to 0.45, *P*=0.379)
**Cardiovascular outcomes**						
Δ Systolic blood pressure	–0.67 (–2.41 to 1.65, *P*=0.717)	–0.27 (–2.60 to 0.79, *P*=0.295)	–0.07 (–2.72 to 2.88, *P*=0.955)	–1.58 (–9.19 to 2.26, *P*=0.236)	–0.96 (–5.50 to 2.39, *P*=0.440)	–3.83 (–8.96 to 2.03, *P*=0.217)
Δ Diastolic blood pressure	–0.21 (–1.40 to 1.47, *P*=0.960)	–0.21 (–1.44 to 0.73, *P*=0.522)	0.14 (–1.09 to 2.89, *P*=0.486)	–1.65 (–7.99 to 0.01, *P*=0.050)	–0.50 (–4.22 to 0.90, *P*=0.203)	–1.16 (–5.31 to 2.05, *P*=0.386)
Δ Total cholesterol	–2.83 (–6.40 to 1.18, *P*=0.178)	–0.08 (–3.56 to 2.49, *P*=0.729)	0.05 (–5.07 to 4.06, *P*=0.828)	–1.25 (–12.78 to 9.96, *P*=0.809)	–1.31 (–11.57 to 1.94, *P*=0.162)	3.18 (–7.62 to 9.36, *P*=0.841)
Δ Triglyceride	–2.72 (–19.17 to 11.05, *P*=0.599)	4.69 (–9.21 to 15.98, *P*=0.598)	–0.90 (–14.21 to 11.33, *P*=0.825)	–13.03(–83.67 to 24.00, *P*=0.277)	8.71 (–17.02 to 49.47, *P*=0.339)	–1.56 (–27.00 to 15.06, *P*=0.578)
ΔLDL cholesterol	–1.53 (–5.00 to 1.89, *P*=0.376)	–0.70 (–3.65 to 1.84, *P*=0.517)	0.92 (–2.72 to 5.08, *P*=0.554)	0.07 (–7.50 to 11.66, *P*=0.671)	–0.07 (–10.60 to 1.52, *P*=0.142)	2.60 (–8.30 to 7.20, *P*=0.889)
**Healthcare utilization outcomes**						
Δ Outpatient frequency	0.20 (–1.81 to 0.98, *P*=0.563)	1.29 (–0.73 to 3.44, *P*=0.203)	–0.50 (–6.83 to 4.59, *P*=0.700)	0.95 (–2.59 to 5.29, *P*=0.503)	–1.20 (–7.04 to 1.07, *P*=0.149)	–2.38 (–10.57 to 5.31, *P*=0.516)
Δ Outpatient cost	39.5 (–283.3 to 222.3, *P*=0.563)	64.6 (–281.9 to 666.5, *P*=0.427)	114.9 (–590.8 to 550.7, *P*=0.563)	86.5 (–463.5 to 181.2, *P*=0.391)	252.2 (–737.8 to 959.1, *P*=0.798)	–879.4 (–2739.8 to –259.4, *P*=0.018)
Δ Inpatient frequency	0.02 (–0.11 to 0.07, *P*=0.679)	–0.03 (–0.12 to 0.15, *P*=0.846)	0.01 (–0.34 to 0.30, *P*=0.898)	0.12 (–0.07 to 0.35, *P*=0.200)	–0.01 (–0.34 to 0.28, *P*=0.830)	–0.31 (–1.11 to 0.19, *P*=0.167)
Δ Inpatient cost	69.2 (–848.5 to 790.8, *P*=0.945)	–218.0 (–880.8 to 1176.3, *P*=0.778)	522.9 (–1512.3 to 2232.2, *P*=0.706)	985.8(–1001.2 to 2561.4, *P*=0.391)	–745.7 (–4525.1 to 492.1, *P*=0.115)	–2462 (–7835.3 to 261.1, *P*=0.067)
Δ Length of stay	0.07 (–1.58 to 1.10, *P*=0.726)	–0.15 (–1.03 to 1.99, *P*=0.536)	0.84 (–2.97 to 4.34, *P*=0.713)	1.26 (–1.53 to 3.31, *P*=0.471)	0.63 (–4.95 to 7.46, *P*=0.691)	–7.91 (–18.81 to 2.22, *P*=0.122)

The sample comprises people within optimal bandwidth of the threshold 0 (SWC: men 85 cm and women 90 cm) and 25 kg/m^2 (BMI). Each cell represents the coefficient with 95% confidence interval from a separate regression. In all regressions, we used a triangular kernel function, which gives more weight to observations closer to the thresholds. All models in these analyses include demographic (age and sex), socioeconomic (income and occupation), and behavioral covariates (smoking, alcohol drinking, regular exercise, and dietary habits). Baseline SWC and BMI in 2015 are used as assignment variables. Abbreviations: SWC, standardized waist circumference; BMI, body mass index; LDL, low-density lipoprotein.

Table S4. Effect of the lifestyle guidance intervention on changes in health behaviors

Changes of health behaviors	SWC	BMI
Smoke	0.98 (0.91 to 1.06, *P*=0.678)	0.84 (0.67 to 1.04, *P*=0.113)
Drink alcohol	0.98 (0.86 to 1.12, *P*=0.768)	0.99 (0.76 to 1.30, *P*=0.973)
Regular Exercise	1.01 (0.94 to 1.09, *P*=0.779)	1.05 (0.89 to 1.24, *P*=0.581)
Dietary habits		
Eating speed	0.92 (0.88 to 0.95, *P*<0.001)	0.90 (0.83 to 0.97, *P*=0.009)
Supper after dinner	0.83 (0.80 to 0.85, *P*<0.001)	0.94 (0.89 to 0.99, *P*=0.027)
Supper before bed	0.83 (0.81 to 0.85, *P*<0.001)	0.94 (0.88 to 0.99, *P*=0.026)
Skipping breakfast	0.84 (0.81 to 0.86, *P*<0.001)	0.94 (0.89 to 0.99, *P*=0.031)
Enough sleep	1.09 (0.96 to 1.11, *P*=0.377)	1.08 (0.92 to 1.27, *P*=0.372)

Baseline SWC and BMI used as assignment variables separately. We used the modified Poisson regression approach to estimate the outcomes of change in health behaviors. Bandwidth of 8 cm from the threshold of SWC and 2 from the threshold of BMI was applied in this regression approach. Each cell represents the relative risk with 95% confidence interval and *P*-value from a separate regression. Abbreviations: SWC, standardized waist circumference; BMI, body mass index.

Table S5. Robustness check for bandwidth selection in the regression discontinuity design analysis

Bandwidth selection	Percentage of optimal bandwidth	SWC		BMI	
		Sample in bandwidth	Impact of outcomes using baseline SWC	Sample in bandwidth	Impact of outcomes using baseline BMI
Δ Weight	50%	14,306	-0.30 (-0.53 to -0.06, *P*=0.015)	2,937	0.07 (-0.44 to 0.70, *P*=0.657)
	80%	22,852	-0.30 (-0.48 to -0.13, *P*=0.001)	4,781	0.15 (-0.35 to 0.47, *P*=0.403)
	120%	31,134	-0.30 (-0.44 to -0.15, *P*<0.001)	7,091	0.19 (-0.18 to 0.46, *P*=0.403)
	150%	42,062	-0.31 (-0.43 to -0.17, *P*<0.001)	8,234	0.14 (-0.07 to 0.50, *P*=0.143)
Δ Waist circumference	50%	14,306	-0.19 (-0.59 to 0.11, *P*=0.174)	2,937	0.12 (-0.70 to 0.86, *P*=0.834)
	80%	22,852	-0.18 (-0.46 to 0.06, *P*=0.124)	4,781	0.16 (-0.45 to 0.69, *P*=0.679)
	120%	31,134	-0.15 (-0.42 to 0.00, *P*=0.052)	7,091	0.17 (-0.29 to 0.63, *P*=0.473)
	150%	42,062	-0.13 (-0.38 to 0.00, *P*=0.044)	8,234	0.13 (-0.22 to 0.63, *P*=0.340)
ΔBMI	50%	14,306	-0.10 (-0.39 to -0.02, *P*=0.019)	2,937	0.03 (-0.14 to 0.26, *P*=0.563)
	80%	22,852	-0.10 (-0.17 to -0.04, *P*=0.001)	4,781	0.05 (-0.11 to 0.18, *P*=0.668)
	120%	31,134	-0.10 (-0.16 to -0.05, *P*<0.001)	7,091	0.06 (-0.06 to 0.17, *P*=0.383)
	150%	42,062	-0.10 (-0.14 to -0.05, *P*<0.001)	8,234	0.05 (-0.03 to 0.18, *P*=0.164)

To test the robustness of bandwidth selection in the regression discontinuity design, we used different bandwidths (50%, 80%, 120%, and 150% of the optimal bandwidth) with robust bias-corrected 95% confidence intervals. The findings were quantitatively unaffected by the use of different bandwidths. Abbreviations: SWC, standardized waist circumference; BMI, body mass index.
